# Epidemiology, Genetic Evolution, and Capsid Protein Variation of Porcine Circovirus 2 in China (2023–2024): Sustained Dominance of Genotype PCV2d

**DOI:** 10.3390/v18040468

**Published:** 2026-04-15

**Authors:** Ze Tong, Shiting Ni, Jiaqi Liu, Pingxuan Liu, Daisheng Shi, Guosheng Chen, Xin Zong, Yaning Lv, Renhang Xiao, Chen Tan

**Affiliations:** 1 National Key Laboratory of Agricultural Microbiology, College of Veterinary Medicine, Huazhong Agricultural University, Wuhan 430070, China; 2 Hubei Hongshan Laboratory, Wuhan 430070, China; 3 Key Laboratory of Preventive Veterinary Medicine in Hubei Province, The Cooperative Innovation Center for Sustainable Pig Production, Wuhan 430070, China

**Keywords:** PCV2, PRRSV, ORF2 gene, capsid protein, viral co-infection, epitope

## Abstract

Porcine circovirus type 2 (PCV2) is a pathogen of major importance in swine that is characterized by ongoing genetic evolution. To provide an updated epidemiological assessment for China, our study analyzed 1051 clinical samples collected from 27 provincial-level regions between 2023 and 2024. The overall PCV2 positivity rate was 65.18%, with detection rates showing significant seasonal variation, with higher rates in spring and summer. Genotypic analysis of 379 open reading frame 2 (ORF2) sequences identified PCV2d as the dominant genotype (78.89%), and no significant geographic clustering was observed. Coinfection with porcine reproductive and respiratory syndrome virus (PRRSV) is common, yet statistical tests have revealed an epidemiologically independent relationship between the two viruses. Notably, analysis of the capsid (Cap) protein revealed that high-frequency amino acid mutations were concentrated in immunodominant loop regions. These mutations resulted in genotype-specific substitutions within key neutralizing epitopes. This study provides the latest large-scale national baseline data on PCV2 in China for 2023–2024. It systematically analyzes the epidemiological characteristics of the dominant PCV2d genotype in the post-African Swine Fever era, the patterns of antigenic epitope mutations in the Cap protein, and their potential impact on vaccine efficacy. The study fills a gap in recent national epidemiological data on PCV2 in China and provides a basis for the targeted prevention and control of PCV2 and the updating of vaccine strains.

## 1. Introduction

Porcine circovirus type 2 (PCV2) is the primary etiological agent of PCV-associated diseases (PCVADs), a group of conditions, including postweaning multisystemic wasting syndrome (PMWS) and porcine dermatitis and nephropathy syndrome (PDNS), which cause considerable economic losses to the global swine industry [1,2,3].

The virus is characterized by a high rate of genetic evolution, leading to the emergence and replacement of different genotypes over time. Since approximately 2012, PCV2d has displaced PCV2b as the predominant genotype in many swine-producing regions worldwide [4]. This genotypic shift is of scientific and practical importance because current commercial vaccines are primarily based on the PCV2a or PCV2b genotype. The accumulation of mutations in open reading frame 2 (ORF2), which encodes the immunodominant capsid (Cap) protein, particularly in circulating PCV2d strains, may affect the cross-protective efficacy of existing vaccines [5].

The effectiveness of PCV2 vaccines is primarily evaluated based on the key metrics: mitigation of clinical signs associated with PCVAD, most notably PMWS and PDNS; reduction in tissue-associated relative viral loads in vaccinated animals; and improvement in production performance parameters, including survival rate and average daily weight gain. It is important to emphasize that the core purpose of PCV2 vaccination is the control of clinical PCVAD manifestation and the reduction in associated economic losses, rather than the achievement of viral eradication or the induction of sterilizing immunity.

The clinical expression of PCVAD can be influenced by coinfections with other swine pathogens, such as porcine reproductive and respiratory syndrome virus (PRRSV). The interaction between PCV2 and PRRSV is not fully understood. Some experimental studies have reported synergistic effects, where coinfection leads to increased viral replication and more severe disease [6,7,8]. Furthermore, recent epidemiological surveillance has highlighted the significant economic impact of different PRRSV infection statuses in breeding pig farms, underscoring the importance of understanding viral co-circulation patterns [9]. In contrast, other studies have suggested that these two viruses may infect swine populations independently, which could be related to the infection order of the two viruses or the immune status of the host [10,11].

As the world’s largest swine producer, China’s pig population is under constant pressure from PCV2. Continuous surveillance is necessary to monitor the evolving epidemiology of the virus. Although several molecular epidemiological studies of PCV2 have been reported in China, the majority of these investigations were conducted using samples collected prior to the 2020 African Swine Fever (ASF) outbreak. Furthermore, many of these prior studies are constrained by limited sample sizes or by restricted geographical coverage confined to single provinces or regions, and consequently may not accurately capture the nationwide epidemiological landscape in the post-ASF era. Meanwhile, recent studies on the mutational landscape of the Cap protein in currently circulating strains, and its coinfection patterns with PRRSV, are also limited.

For these reasons, the present study was conducted to provide an updated molecular and epidemiological analysis of PCV2 in China between 2023 and 2024. The objectives of this study were to (1) determine the prevalence, seasonal distribution, and genotypic composition of PCV2 across different regions; (2) analyze amino acid mutations in the Cap protein, focusing on their positions relative to known antigenic sites; and (3) evaluate the coinfection frequency of PCV2 and PRRSV to assess their epidemiological relationship. The results are intended to provide current data for informing vaccine development and disease control measures in China.

## 2. Materials and Methods

### 2.1. Sample Collection and Processing

From 2023–2024, a total of 1051 tissue samples were collected from swine herds across 27 provincial-level regions in China. The tissues collected were to lymph nodes (superficial inguinal or mesenteric) and lung tissue; no kidney, liver, spleen, or other organ tissues were included. These samples originated from pigs exhibiting clinical signs consistent with PCVADs. The tissue samples were homogenized in sterile phosphate-buffered saline (PBS) and centrifuged at 9000× *g* for 3 min at 4 °C. The resulting supernatants were collected and stored at −80 °C until nucleic acid extraction.

### 2.2. Nucleic Acid Extraction and Virus Detection

Total viral DNA and RNA were coextracted from 200 μL of tissue supernatant via a magnetic bead-based kit (Vazyme, RM401, Nanjing, China). PCV2 and PRRSV were detected via real-time qPCR on a MA-688 real-time PCR detection system (Molarray, Suzhou, China). PCV2 DNA was detected via a TaqMan probe-based qPCR assay with AceQ Universal U+ Probe Master Mix (Vazyme, Q513, Nanjing, China). The thermal profile included a UDG digestion step at 37 °C for 2 min and predenaturation at 95 °C for 5 min, followed by 40 cycles of 95 °C for 10 s and 60 °C for 30 s (Table 1). PRRSV RNA was detected via a one-step qRT-PCR assay with the HiScript II U+ One Step qRT-PCR Probe Kit (Vazyme, Q223, Nanjing, China). The thermal profile consisted of reverse transcription at 55 °C for 15 min and pre-denaturation at 95 °C for 3 min, followed by 40 cycles of 95 °C for 30 s and 55 °C for 30 s.

### 2.3. PCV2 ORF2 Gene Amplification and Sequencing

From the qPCR-positive samples, the complete open reading frame 2 (ORF2) gene was amplified from PCV2-positive samples via high-fidelity PCR with 2× Hieff Canace Plus PCR Master Mix (YeaSen, 10154, Shanghai, China). Samples that failed to yield sufficient ORF2 amplicon were not processed further. The PCR program included initial denaturation at 98 °C for 3 min, followed by 35 cycles of 98 °C for 10 s, 55 °C for 20 s, and 72 °C for 30 s, with a final extension step. The purified PCR amplicons were subjected to bidirectional Sanger sequencing, and sequences that did not meet quality criteria (ambiguous base calls, incomplete ORF2, or frameshifts) were discarded.

### 2.4. Phylogenetic and Sequence Analyses

The obtained ORF2 sequences were assembled and edited via SnapGene software v3.2.1 (GSL Biotech, Kothrud, Pune, India). For phylogenetic analysis, sequences were aligned with representative PCV2 reference sequences (genotypes a–i) from GenBank via the ClustalW method in MEGA V12 software. A neighbor-joining phylogenetic tree was constructed on the basis of the complete ORF2 coding sequences to determine the genotype of each field strain. The phylogenetic tree was visualized and annotated via iTOL v7.2.1.

### 2.5. Capsid Protein Mutation and Structural Analysis

The ORF2 nucleotide sequences were translated into their corresponding Cap protein amino acid sequences via DNASTAR Lasergene software v7.1. Amino acid mutations and sequence conservation were analyzed via MEGA12 and visualized via GeneDoc. The 3D structure of a representative Cap protein monomer was predicted with AlphaFold 2 and rendered in PyMOL v3.1. Linear B-cell epitopes were predicted via the BepiPred-3.0 server, and potential N-linked glycosylation sites were identified via the NetNGlyc-1.0 server. Known neutralizing epitopes from the Immune Epitope Database and published literature were mapped onto the protein structure.

### 2.6. Statistical Analysis

All the statistical analyses were performed via R software (v4.5.1). The associations between categorical variables were evaluated via the chi-square (*χ*^2^) test. Monotonic trends in seasonal prevalence were assessed with the Cochran-Armitage trend test. For coinfection analysis, Pearson residuals were calculated from a 2 × 2 contingency table to measure the deviation from expected frequencies under the hypothesis of independence. Differences in relative viral loads (expressed as Ct values) between the single-infected and coinfected groups were compared via the nonparametric Mann-Whitney *U* test. The associations between PCV2 genotypes and geographic regions were analyzed via Fisher’s exact test, supplemented with 10,000 Monte Carlo simulations to handle small expected cell frequencies. Data visualizations were created via R packages (e.g., ggplot2), dycharts and WebLogo3.

## 3. Results

### 3.1. PCV2 Prevalence in China Is High and Seasonally Dependent

**Figure 1:**
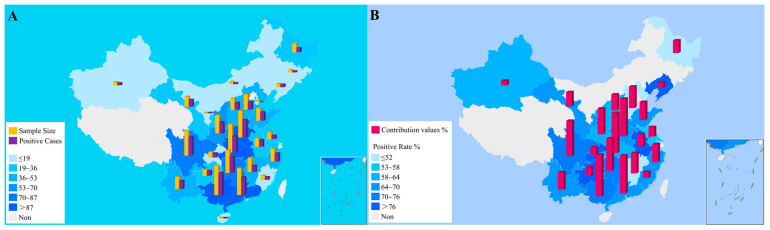
Geographic distribution and statistical analysis of PCV2 positivity rates in China. (**A**) Original spatial distribution of PCV2-positive cases and sample collection data. (**B**) PCV2 positivity rates and adjusted positivity rates after data normalization.

To establish the current epidemiological status of PCV2 in China, comprehensive surveillance was conducted from 2023–2024. A total of 1051 tissue samples were collected from clinically affected swine herds across 27 provincial-level regions, with 685 samples tested positive for PCV2 via real-time PCR (Figure 1A). To account for the impact of provinces with small sample sizes on the statistical analysis results, data normalization was employed. After excluding regions with fewer than 10 samples for robust statistical analysis, 670 of the remaining samples tested positive, yielding an overall prevalence of 65.30% (670/1026). Geographically, PCV2 was widespread, with a particularly high prevalence observed in the high-density swine-producing provinces of Southwest, South, East and Central China. For example, provinces such as Guizhou (80.00%), Guangxi (74.23%), Anhui (82.14%) and Hunan (70.67%) presented positivity rates significantly above the national average (Figure 1B).

**Figure 2:**
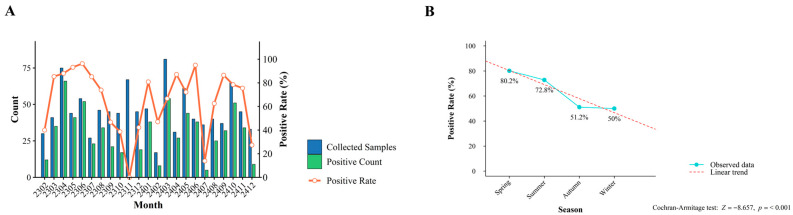
Seasonal Dynamics of PCV2 Positivity Rates in China. (**A**) Monthly variation in PCV2 positivity rates. (**B**) Declining trend of PCV2 positivity via the Cochran-Armitage test.

The prevalence of PCV2 also exhibited a seasonal variation in the observed data. Positivity remained elevated across all seasons, exceeding 50% throughout the year. Spring exhibited the highest detection rate (80.18%), followed by summer (72.84%), while autumn and winter showed comparatively lower but still substantial rates (>50%) (Figure 2A). Standardized residual analysis indicated a positive deviation from expected values in spring, whereas in autumn, it was a negative deviation. Furthermore, a Cochran-Armitage trend test yielded a statistically monotonic decrease across the four seasons (Figure 2B).

### 3.2. PCV2 and PRRSV Circulate as Independent Pathogens

**Figure 3:**
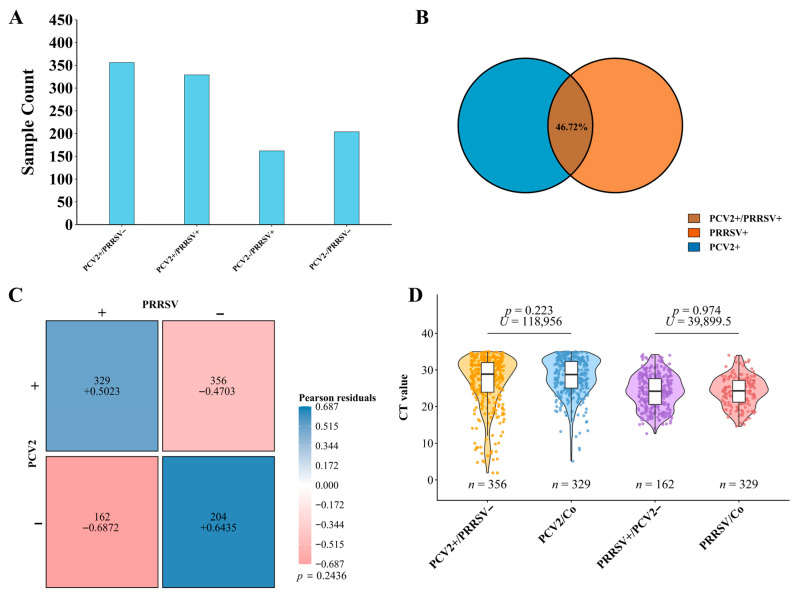
Epidemiological patterns of PCV2 and PRRSV coinfection (2023–2024). (**A**) Prevalence of PCV2 and PRRSV. (**B**) Coinfection rates of PCV2 and PRRSV. (**C**) Pearson residual analysis of infection patterns. (**D**) Relative viral load comparison between the single infection and coinfection groups.

To investigate the epidemiological relationship between PCV2 and PRRSV, all 1051 valid samples were tested for both viruses. The overall PRRSV prevalence was 46.72% (491/1051), and a substantial portion of the samples (31.30%, 329/1051) were positive for both viruses (Figure 3A,B). To determine whether this co-occurrence was statistically significant or merely a result of high individual prevalence, we analyzed the association between the two infections. The chi-square test revealed no significant associations (*χ*^2^ = 1.36, *p* = 0.24 > 0.001). This lack of association was further supported by Pearson residual analysis, where the observed frequency of coinfection did not significantly deviate from the expected frequency under a model of independence (residual for PCV2+/PRRSV+ = +1.17) (Figure 3C).

To explore potential interactions at the viral replication level, we compared relative viral loads (expressed as Ct values) between the single-infected and coinfected groups. It should be noted that Ct values represent an indirect, relative indicator of viral nucleic acid abundance and do not constitute a direct measurement of absolute viral load. Lower Ct values correspond inversely to higher initial viral template concentrations in the sample, signifying higher relative viral burden. The analysis revealed no statistically significant difference in PCV2 relative viral loads between the two groups (median *Ct* 28.76 vs. 28.87; Mann–Whitney *U* = 118,956, *p* = 0.223), nor in PRRSV relative viral loads (median *Ct* 24.30 vs. 24.20; Mann–Whitney *U* = 39,899.5, *p* = 0.974) (Figure 3D). Collectively, the analysis of both infection frequency and relative viral load provides strong evidence that PCV2 and PRRSV cocirculate as epidemiologically independent pathogens in the field without apparent synergistic or antagonistic effects at the population level.

### 3.3. PCV2d Is the Dominant Genotype Nationwide

**Figure 4:**
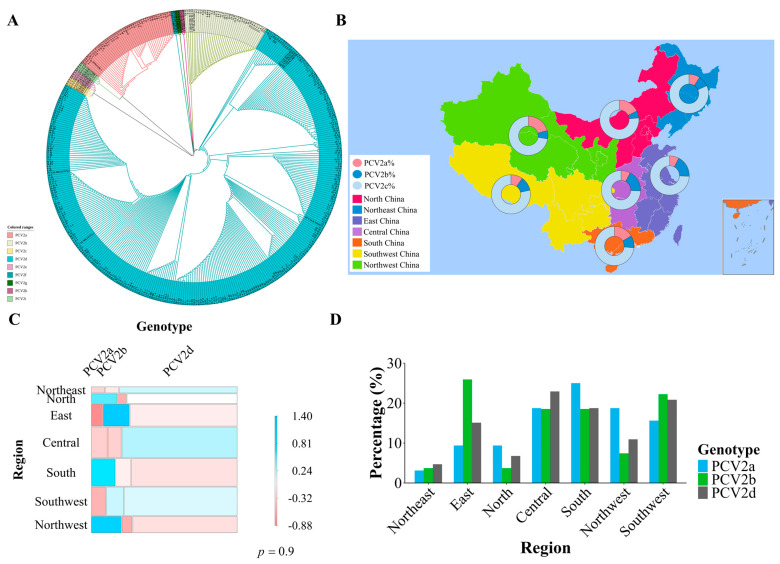
Geographic distribution and genetic evolution of PCV2 genotypes. (**A**) Evolutionary tree of the PCV2 ORF2 gene. (**B**) Genotype composition of PCV2 across seven geographic regions. (**C**) Heatmap of Pearson residuals for genotype distribution. (**D**) Regional contribution to national genotype detection.

To characterize the genetic landscape of PCV2 in China, the ORF2 gene, which encodes the immunodominant Cap protein, was sequenced from 379 positive samples. Phylogenetic analysis of these sequences revealed a clear genotypic structure. The vast majority of strains, 78.89% (299/379), belonged to the PCV2d genotype. The historically significant genotypes PCV2a (11.87%, 45/379) and PCV2b (9.23%, 35/379) were also present but at much lower frequencies. Notably, no other genotypes, including the more recently described PCV2i, were detected in this extensive sampling (Figure 4A).

Geographically, PCV2d was the dominant genotype in all seven major regions of China, with its proportion ranging from 81.82% in North China to 72.41% in Northwest China (Figure 4B). While some minor regional variations were noted, such as a relatively high proportion of PCV2b in East China (17.95%) and PCV2a in Northwest China (20.69%), Pearson’s residual analysis confirmed that there was no significant overall geographic clustering of genotypes (*p* = 0.90) (Figure 4C). These results demonstrate that PCV2d has begun to replace previous genotypes nationwide, and is becoming the dominant circulating strain across China (Figure 4D).

### 3.4. PCV2 Capsid Protein Exhibits Significant Antigenic Drift in Key Epitopes

**Figure 5:**
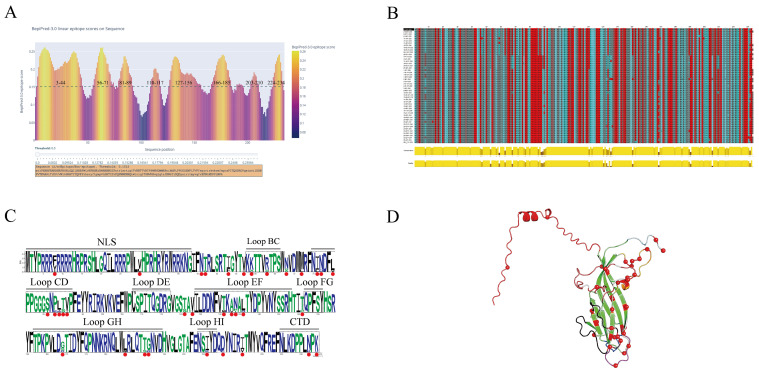
Bioinformatic characterization of the PCV2 Cap protein. (**A**) Antigenicity prediction of the Cap protein via BepiPred. (**B**) Sequence alignment and conservation analysis of the Cap protein (red and blue represent different types of amino acids). (**C**) LOGO plot of amino acid mutation sites (black and green represent different types of amino acids, and red indicates where mutations occur). (**D**) 3D structure of the Cap protein monomer predicted by AlphaFold.

To investigate the molecular evolution driving the success of PCV2d, the amino acid sequences of 379 Cap proteins were analyzed for mutational patterns after the sequences were removed, with a focus on functionally and antigenically important regions. The analysis identified 34 polymorphic sites across all the sequences. Strikingly, 61.8% (21/34) of these mutations were concentrated within the structurally exposed loop regions, which correspond to predicted zones of high antigenicity (Figure 5). Loop CD (residues 75–92), which forms part of the capsid’s twofold axis, was a particular mutational hotspot, with 44.4% of its sites mutated.

**Figure 6:**
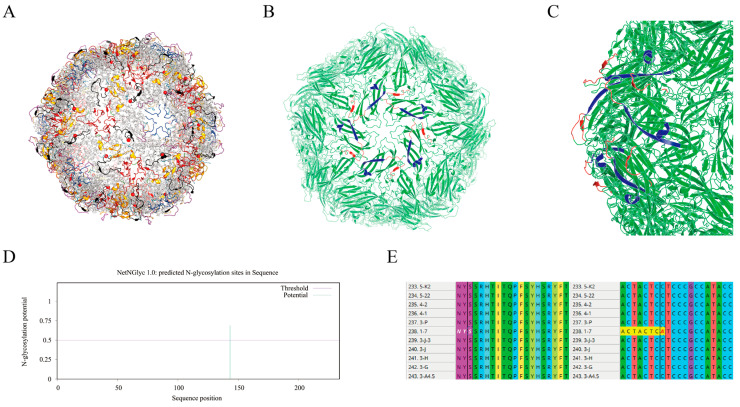
Structural features and N-linked glycosylation analysis of the PCV2 viral particle and Cap protein. (**A**) Glycosylation sites and loops and axial systems of PCV2 viral particles. (**B**,**C**) Location of the neutralizing epitope of the PCV2 viral particle. (**D**) Prediction of N-linked glycosylation sites in the PCV2 Cap protein. (**E**) Conservation analysis of N-linked glycosylation sites in the PCV2 Cap protein (different colors represent different types of amino acids and nucleotides).

**Figure 7:**
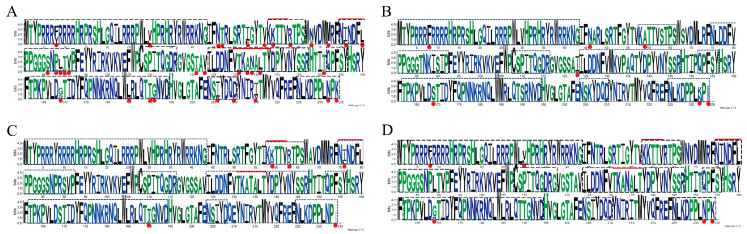
LOGO Plots of Amino Acid Mutations Across PCV2 Genotypes. (**A**) Sequence alignment of all the genotypes. (**B**) Alignment of PCV2a Cap protein sequences. (**C**) Alignment of PCV2b Cap protein sequences. (**D**) Alignment of PCV2d Cap protein sequences. The black and green represent different types of amino acids, and red indicates where mutations occur.

**Figure 8:**
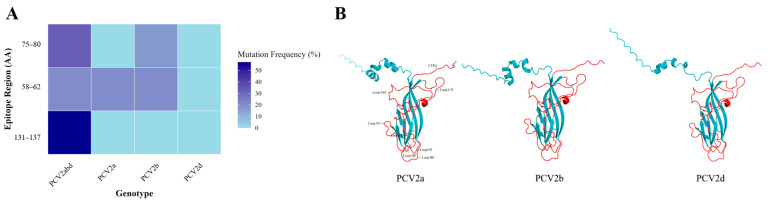
LOGO Plots of Cap Protein Structures Across PCV2 Genotypes. (**A**) Neutralization epitope mutation frequency of the PCV2 Cap protein. (**B**) Cap protein structure.

When we focused on known neutralizing epitopes, a key conformational epitope within Loop EF (residues 131–137) exhibited a high mutation frequency of 57.1% (4/7 sites). This region is a known target for potent neutralizing antibodies. In stark contrast, a predicted N-linked glycosylation site (143-NYSS-146), also located in Loop EF, was 100% conserved at both the amino acid and nucleotide levels across all 379 strains, suggesting strong purifying selection (Figure 6, Table 2). Furthermore, a detailed comparison revealed that while the intragenotype diversity was low (only 5–7 polymorphic sites within each of PCV2a, 2b, and 2d), numerous consistent amino acid substitutions differentiated the three genotypes. Many of these genotype-defining mutations, such as those at positions 59, 89, and 134, are located within or near key antigenic regions (Figure 7, Figure 8, Table 3). These findings provide potential molecular evidence of significant antigenic drift in the PCV2 Cap protein and pinpoint the specific epitope regions that differentiate the dominant PCV2d strains from the older PCV2a/b vaccine strains, thus offering a molecular explanation for potential vaccine mismatches. However, the actual effects of these mutations still need to be further verified through animal experiments.

## 4. Discussion

Our comprehensive molecular and epidemiological surveillance of Porcine Circovirus Type 2 (PCV2) in China from 2023–2024 offers a critical update on its status. This investigation confirmed the high prevalence of the virus in symptomatic swine, documented the enriched dominance of the PCV2d genotype, clarified the relationship of the virus with PRRSV, and revealed key evolutionary trends in the capsid (Cap) protein that directly impact vaccine efficacy.

The overall PCV2 positivity rate of 65.18% in clinically affected herds underscores its persistent and widespread circulation within Chinese swine populations. This prevalence, comparable to that reported in other recent large-scale surveys, indicates that despite widespread vaccination, PCV2 remains a major contributor to swine diseases [12]. We believe this may be the result of a combination of the following factors: First, most commercial vaccines are based on PCV2a or PCV2b genotypes and confer robust but incomplete cross-protection against the predominant PCV2d strain. Second, high-density farming practices in China amplify within-herd and between-herd transmission pressure. Third, current vaccines effectively reduce clinical disease and viral shedding but do not induce sterilizing immunity; vaccinated pigs may continue intermittent shedding via oronasal and fecal routes, sustaining viral circulation within herds. Critically, the results of the present study confirm that PCV2d is the dominant genotype (78.89%) across all major regions of China, confirming the global trend of its replacement of the former PCV2b genotype [13,14]. A lack of significant geographic clustering suggests that PCV2d is now homogeneously distributed, likely facilitated by extensive swine movement. Vaccine-induced immune pressure from long-term use of PCV2a- and PCV2b-based products is widely considered the primary driver for this genotypic shift, as such pressure selectively favors the proliferation of antigenically distinct variants such as PCV2d [15,16].

A key finding from our analysis is the epidemiologically independent coinfection pattern between PCV2 and PRRSV. Although coinfection was frequent (31.30%), statistical analysis of both infection status and relative viral loads revealed no evidence of a synergistic or antagonistic relationship at the population level. This observation contrasts with experimental studies in which PRRSV pre-infection has been shown to exacerbate PCV2 replication [7,8,17]. Results from laboratory-based virulence investigations often diverge significantly from epidemiological patterns observed in the field which is a challenge that has long vexed researchers, including those involved in our own study. To mitigate this discrepancy as effectively and rationally as possible, we sought the aid of various novel technologies (such as bioinformatics analytical framework, AlphaFold, and more). In particular, the application of a biomedical knowledge graph, a framework designed for the integrated management of diseases and pathogens, enabled us to derive more meaningful interpretations from our data [18]. In such real-world settings, variables such as infection order, host genetics, passive immunity, and the presence of other pathogens can mask direct viral interactions [8]. Therefore, while PRRSV infection may remain a risk factor for PCVAD severity at the individual level, our data suggest that, from a large-scale epidemiological perspective, PCV2 and PRRSV circulate as independent threats. This finding supports a “dual-track” control strategy, where measures targeting each pathogen can be optimized independently.

Our data suggest a potential seasonal influence on PCV2 prevalence. Prior studies in China have similarly reported elevated detection in April–May and reduced detection in December–January, potentially attributable to environmental conditions favoring virion stability or production-related stressors [19]. In contrast, long-term surveillance in the United States has not revealed comparable seasonal trends, highlighting how national production systems may differentially influence viral epidemiology [20]. We recognize sampling limitations, as our core data derive from clinically affected pigs in traditional major production regions. Additionally, variability in the timing of herd immunization and duration of vaccine-induced protection may further modulate seasonal detection patterns. However, we recognize that broader surveillance including asymptomatic herds would be required to fully characterize population-level seasonal dynamics.

Molecular analysis of the Cap protein provides a mechanistic basis for concerns regarding vaccine efficacy, revealing a dual evolutionary strategy: high-frequency mutations are concentrated within immunodominant surface loops, specifically the loop EF epitope (residues 131–137), where a 57.1% mutation rate indicates significant antigenic drift at a key neutralizing antibody target [21,22]. The adjacent N-linked glycosylation site (143-NYSS-146) remains fully conserved, likely maintaining viral fitness through a stable glycan shield. It is noteworthy that the magnitude of cross-protection conferred by existing PCV2a/b-based vaccines against PCV2d field strains is not uniform but rather appears to be contingent upon the specific repertoire of mutations present within immunodominant epitopes. The high-frequency mutations identified within the Loop EF neutralizing epitope in this study represent a region warranting prioritized investigation in subsequent functional validation studies, as this domain constitutes one of the principal determinants governing heterologous vaccine cross-protective efficacy. Furthermore, consistent amino acid substitutions that define each genotype underscore substantial antigenic divergence between circulating PCV2d strains and older PCV2a/b vaccine strains. This expanding genetic and antigenic gap may provide molecular evidence for evaluating the cross-protective efficacy of existing PCV2a/b vaccines against the PCV2d strain. However, the actual impact of these mutations on vaccine-induced protective immunity remains to be empirically determined through in vitro virus neutralization assays and in vivo animal challenge experiments.

Our study has several limitations. The primary limitation resides in the inherent selection bias associated with the sampling strategy. All specimens analyzed herein were collected exclusively from pigs exhibiting clinical signs consistent with PCVAD. Therefore, the observed PCV2 positivity rate of 65.18% cannot be extrapolated to represent the overall infection prevalence within the broader Chinese swine population, nor does it reflect the viral carriage rate among asymptomatic or subclinical infected herds. This targeted sampling of symptomatic animals likely results in a certain degree of overestimation of the true PCV2 prevalence at the national herd level. Second, the coinfection analysis was limited to PRRSV. Third, reliance on ORF2 sequencing, while standard for genotyping, cannot detect genome-wide recombination or mutations in other functional regions. Future research employing whole-genome sequencing and broader sampling strategies would provide a more complete epidemiological picture.

## 5. Conclusions

In conclusion, this investigation documents the dominance of the PCV2d genotype in China and demonstrates its ongoing antigenic evolution, particularly within key neutralizing epitopes of the capsid protein. The finding of an independent epidemiological relationship with PRRSV suggests that control strategies can effectively target these pathogens separately at the population level. The extensive amino acid polymorphisms identified within the Cap protein epitopes of circulating PCV2d strains provide a molecular reference for evaluating the cross-protective efficacy of existing commercial vaccines and for informing future vaccine strain optimization strategies. Continuous molecular surveillance remains essential for monitoring the dynamic evolution of PCV2 and informing future control measures.

## Figures and Tables

**Table 1:** Primers and probes used in PCV2 and PRRSV DNA detection.

Primer	Sequence
PCV2-F	5′-CCCTGTCACCCTGGGTGAT-3′
PCV2-R	5′-CCTGTGCCTTTGAATACTACAGA-3′
PCV2-Probe	5′-FAM-TAAGGTTGAATTCTGGCCCTGCTCCC-BHQ1-3′
PRRSV1-F	5′-CAGATGCAGATTGTGTTGCCT-3′
PRRSV1-R	5′-ATGGAGACCTGCAGCACTTTC-3′
PRRSV2-F	5′-TTGTGCTTGCTAGGCCGC-3′
PRRSV2-R	5′-ACGACAAATGCGTGGTTATCA-3′

**Table 2:** Mutation statistics of loops and neutralization epitopes in the PCV2 Cap protein.

Loops	Loop BC 58–66	Loop CD 75–92	Loop DE 108–117	Loop EF 124–149	Loop FG 154–158	Loop GH 164–196	Loop HI 206–210	CTD 227–236	
Ratio	2/9	**8/18**	0	6/26	0	4/33	1/5	2/10	
Rate	22.22%	**44.44%**		23.07%		12.12%	20%	20%	
Neutralization Epitope	47–58	55–56	58–62	75–80	88	128	**131–137**	189	231
Ratio	3/12	0	1/5	2/6	1	0	**4/7**	0	0
Rate	25%		20%	33.33%			**57.14%**		

The bolded data in table represent the regions with the high mutation frequency in the loops and neutralization epitopes in the PCV2 Cap protein.

**Table 3:** Amino acid mutation sites in cap proteins across PCV2 genotypes.

AA Genotypes	8	30	46	47	53	57	59	63	68	72	76	77	80	86	88	89	90
All	F/Y	V/L	N/S	T/A	I/F	V/I	K/A/R	R/S/K	N/S/A	M/L	I/L	N/D	L/V	S/T	P/K	L/I/R	T/S
PCV2a	Y	V	N/S	A	F	V	A	S	S	L	L	D	V	T	K	I	S
PCV2b	F	V	N	T	F	I	K/R	R/K	A	M	I/L	N	L	S	P	R	S
PCV2d	F/Y	V/L	N	T	I	V	K	R	N	M	I	N	L	S	P	L	T
**AA** **Genotypes**	**91**	**121**	**123**	**131**	**133**	**134**	**136**	**153**	**171**	**187**	**192**	**193**	**208**	**212**	**217**	**234**	**236**
All	V/I	T/S	V/I	T/M	A/V	N/P/T	L/Q	T/P	G/S/R	L/M	T/S/A	G/R	I/K	D/E	I/V	N/K	K/Q/-
PCV2a	I	S	V/I	M	V	P	Q	P	S	M	S	R	K	D	V	N/K	K/-
PCV2b	V	S	V	T	A	T	L	P	S	L	T/A	G	I	E	V	N	K/-
PCV2d	V	T	I	T	A	N	L	T	G/R	L	T	G	I	D	I	N/K	K/Q

## Data Availability

The original contributions presented in this study are included in the article and Supplementary Materials. Further inquiries can be directed to the corresponding author.

## Supplementary Materials

The following supporting information can be downloaded at: https://www.mdpi.com/article/10.3390/v18040468/s1, Table S1: Background information and test results for PCV2 and PRRSV from 1051 tissue samples collected from swine herds; Table S2: 379 ORF sequences and their PCV2 genotype.
